# The Effect of Physical Aging and Degradation on the Re-Use of Polyamide 12 in Powder Bed Fusion

**DOI:** 10.3390/polym14132682

**Published:** 2022-06-30

**Authors:** Benjamin Sanders, Edward Cant, Hoda Amel, Michael Jenkins

**Affiliations:** 1 School of Metallurgy and Materials, University of Birmingham, Elms Road, Birmingham B15 2SE, UK; bls715@student.bham.ac.uk; 2 The Manufacturing Technology Centre, Ansty Park, Coventry CV7 9JU, UK; edward.cant@the-mtc.org (E.C.); hoda.amel@the-mtc.org (H.A.)

**Keywords:** powder bed fusion, polyamide 12, DSC, recyclability, thermo-oxidation, secondary crystallisation

## Abstract

Powder bed fusion (PBF) is an additive manufacturing (AM) technique which offers efficient part-production, light-weighting, and the ability to create complex geometries. However, during a build cycle, multiple aging and degradation processes occur which may affect the reusability of the Polyamide 12 (PA-12) powder. Limited understanding of these phenomena can result in discarding re-usable powder unnecessarily, or the production of parts with insufficient properties, both of which lead to significant amounts of waste. This paper examines the thermal, chemical, and mechanical characteristics of PA-12 via an oven storage experiment that simulates multi jet fusion (MJF) conditions. Changes in the properties of PA-12 powder during oven storage showed two separate, time-dependent trends. Initially, differential scanning calorimetry showed a 4.2 °C increase in melting temperature (T_m_) and a rise in crystallinity (X_c_). This suggests that secondary crystallisation is occurring instead of, or in addition to, the more commonly reported further polycondensation process. However, with extended storage time, there were substantial reductions in T_m_ and X_c_, whilst an 11.6 °C decrease in crystallisation temperature was observed. Fourier transform infrared spectroscopy, a technique rarely used in PBF literature, shows an increased presence of imide bonds—a key marker of thermo-oxidative degradation. Discolouration of samples, an 81% reduction in strength and severe material embrittlement provided further evidence that thermo-oxidative degradation becomes the dominant process following extended storage times beyond 100 h. An additional pre-drying experiment showed how moisture present within PA-12 can also accelerate degradation via hydrolysis.

## 1. Introduction

Polymer powder bed fusion (PBF) is a subset technique in additive manufacturing (AM) which has attracted attention because it offers great design freedom and high efficiency, as parts can be stacked freely in the powder bed [1,2,3,4,5]. Industrial sectors such as medical, aerospace and architecture have benefitted immensely from the use of additive manufacturing and more specifically the PBF technology. This is a result of the advantages the technology offers including customization, light-weighting, and the ability to create complex geometries [6,7,8,9]. The most common PBF technique is laser sintering (LS) [3], but more recently, HP introduced the multi-jet fusion (MJF) process [7]. Both processes involve storing thermoplastic powder at an elevated temperature, close to the melting point, before the localised application of heat to consolidate the material into a 3D part [3]. As per other AM methods, powder is added layer by layer into a geometry fabricated directly from a 3D-CAD model [3,10].

Materials for PBF require a large processing window, in which the powder melting temperature (T_m_) is significantly higher than the crystallisation temperature (T_c_). Maintaining a high temperature in the build volume delays the crystallisation process during a PBF build, which reduces the residual stresses, prevents distortions, and provides a more homogeneous microstructure [11,12,13,14,15]. Due to its large processing window and the ability to form highly crystalline structures, Polyamide 12 (PA-12) is the most widely used polymer in PBF [11,13,14,16,17]. Polyamides are also popular due to their high recyclability, which can be a major cost saving advantage [7,18]. Nonetheless, PBF has a low powder utilisation rate, whereby at least 80% of the powder deposited for each build cycle remains un-sintered [2,7,19,20,21]. For the process to be economically viable and environmentally sustainable, the un-sintered powder needs to be reused [1,2,16]. A key consideration regarding the re-usability of PA-12 powder is ageing of the un-sintered powder during each build. This occurs because the bed chamber is heated to elevated temperatures for extended periods, which can lead to thermal aging processes such as secondary crystallisation [10,20,22] and, in the case of MJF, thermo-oxidative degradation [7,10,11]. The PBF process also includes a slow cool down phase which extends aging of the un-sintered powder [1,2,23]. As a result, the un-sintered, or “used” powder, is “refreshed” with virgin material and used in subsequent builds. The ratio of used to virgin powder is known as the refresh ratio and, although it differs for LS and MJF, typically about 20% to 50% of the used powder is discarded after every build cycle [5,16,17,20,24]. This incurs significant costs and environmental impacts [7].

Furthermore, assuming that the PA-12 powder used in the build contains recovered powder, these ageing processes have the potential to affect the physical properties of final parts. Components made using aged powder are often more porous and brittle [12], whilst the “orange peel” effect is a common surface defect [2,13,20,21]. To limit the number of parts which are discarded due to their insufficient properties, manufacturers often use a higher proportion of new powder than necessary [1,2,12,16,19]. Owing to the high cost of PA-12 [1,16,20], maximizing the re-use of un-sintered powder is economically desirable. Currently, powder recycling protocols do not fully account for any variation in the properties of “used” powder, and refresh ratios are arbitrary [1,2,19]. As such, the quality of refreshed powder mixtures is often inconsistent, or unknown, so the property profile of a final component is difficult to predict [5]. Therefore, a greater understanding of aging and degradation processes is required to help ease current environmental and economic concerns regarding powder waste, which is necessary for proliferation and widespread use of this technology.

Under PBF conditions, at elevated powder bed temperatures, PA-12 may undergo complex aging mechanisms. Storing PA-12 at high temperatures can cause irreversible chemical changes, resulting in a change to the polymer molecular weight (M_w_) [19,21,25]. Polycondensation (also referred to as postcondensation) describes linear macromolecular chain growth through reactions of end groups, resulting in increased M_w_ [16,17,20,25,26]. Additionally, thermo-oxidation often leads to cross-linking and chain scission which can result in opposing effects [5,13,20]. Cross-linking involves the formation of covalent bonds between polymer chains, leading to increased M_w_ [13,19,24], whilst chain scission in the polymer backbone results in a rapid decrease in M_w_ [10,27]. Furthermore, when polyamides are heated to elevated temperatures, the chain mobility in the amorphous regions allows the degree of crystallinity in the polymer to increase. This is known as secondary crystallisation, and it occurs via a slow, but continuous lamellar thickening mechanism [28,29,30]; depending on the temperature, new lamellae can form in the amorphous interlayer [28,31,32]. These processes can affect the behaviour of PA-12 powder, and, subsequently, the properties of final parts. Secondary crystallisation results in an increased T_m_, which may cause incomplete melting, leading to un-molten, nascent particles remaining in the part [33]. Similarly, increases in melt viscosity, due to rising M_w_ or enhanced cross-link density, can cause insufficient powder coalescence [5,10,20,21] and part consolidation [13,25,34,35]. At the powder bed temperature, physical aging and degradation processes may occur simultaneously and interact with each other [10,12]. For example, chain scission alters the morphology of PA-12, which can increase secondary crystallisation through chemi-crystallisation [36,37,38]. Conversely, cross-linking may reduce secondary crystallisation by restricting motion and reorganisation of polymer chains [21]. Clearly, the chemical and physical aging of PA-12 during PBF is a complex problem, and previous work has explored it from a range of perspectives.

Plastic melt flow rate (MFR) has been used previously to characterise the effect of degradation on the melt-flow properties of PA-12 powder [1,2,7,10,16,39]. MFR values have been used to define the refresh ratio [7], and Dotchev and Yusoff concluded that a powder MFR > 26 g 10 min^−1^ will produce a component with sufficient properties [1]. However, the use of MFR as a metric for powder quality is limited in that it only focuses on one material property: melt flowability/viscosity. Differential scanning calorimetry (DSC) has been used in previous studies to explore the thermal aging of PA-12, within the context of PBF processes. Increased T_m_, broadening of the melting region and decreased crystallisation temperature are common observations [7,19,21]. However, there is contradiction within PBF literature regarding the cause of the changes in T_m_. The majority of current studies use polycondensation, and rising M_w_, as an explanation for increased T_m_ [10,13,17,19,20,35,39,40,41,42]. A few papers state that secondary crystallisation may explain the shift in T_m_ [10,20,22], but the possibility of this lamellar thickening process occurring simultaneously is generally overlooked and requires more attention. Previous studies that age powder in situ in LS or MJF systems tend to be limited to relatively short ageing time periods [16,20,26], or only compare the difference between “virgin” and “aged” samples [14,22,34]. Other studies have attempted to replicate LS conditions over extended time periods by storing virgin PA-12 powder in a deoxygenated vacuum oven, set at 170 °C [2,5,12,43]. As MJF is a more recently developed process, studies exploring aging and degradation of PA-12 in a high-temperature, oxygenated environment are far more limited.

Therefore, this study aims to develop current understanding of the aging and degradation processes which occur when PA-12 is stored at an elevated temperature, for a range of extended time periods, in an oxygenated environment. Since Fourier transform infrared spectroscopy (FTIR) has rarely been used as a characterisation technique in the PBF literature, FTIR will be used in conjunction with DSC and mechanical testing to elucidate the ageing processes occurring under simulated MJF conditions.

## 2. Materials and Methods

### 2.1. Characterisation of Virgin PA-12 Powder

Commercial-grade HP high reusability polyamide 12 (PA-12) was supplied by Hewlett-Packard in powder form. Sample variability was assessed by recording the melting point of six unconditioned powder samples, whilst the thermal stability of virgin PA-12 powder was also studied (see Section 2.2.1 for further information).

### 2.2. Oven Conditioning of Virgin PA-12 Powder

The exact temperature of the multi-jet fusion (MJF) bed chamber is confidential, so the storage conditions used within this work were informed by the patent for MJF [44]. To simulate the conditions found within MJF, the as-received, “virgin”, PA-12 powder was stored at a temperature of 170 °C, in an air-circulating Carbolite-Gero PF 60 oven. Storage times were selected to reveal the extent to which the ageing processes could occur and the interplay between them. Virgin powder was stored for up to 336 h, with samples removed at the following time intervals (hours): 24, 48, 72, 96, 120, 144, 168, 192, 226, 264, 310, 336. A minimum of three samples were analysed per storage time to ensure reproducibility of measurements. Following oven conditioning, powder was stored at room temperature and sealed with parafilm to prevent any further degradation.

#### 2.2.1. Thermal Analysis Using Differential Scanning Calorimetry (DSC)

A Mettler Toledo DSC-1, calibrated from the melting behaviour of zinc (T_m_ 419.5 °C, ΔH_f_ 107.5 Jg^−1^) and indium (T_m_ 156.6 °C, ΔH_f_ 28.45 Jg^−1^) and purged under nitrogen gas flow, was used for the thermal characterisation of PA-12. The different DSC procedures for each experimental section are shown in Table 1.

Following oven conditioning, analysis of the thermal response was primarily carried out on the first heating and cooling cycle to study the effect of thermal annealing on PA-12. Aging and degradation processes often cause melting and crystallisation peaks to shift [10,19,20,22,39,43]. The heat of fusion (ΔH_f_) was determined from the melting peak, and this was used to calculate percentage crystallinity (X_c_) using Equation (1), whereby ΔH_f_ = 209.3 Jg^−1^ (100% crystalline PA-12) [17].

(1) $$X_{c}\left(\%\right)=\frac{\Delta H_{f}}{\Delta H_{f}{}^{0}}\times 100$$

#### 2.2.2. Attenuated Total Reflectance−Fourier Transform Infrared Spectroscopy (ATR-FTIR)

Samples were recovered from the DSC pans and characterised using a Nicolet 8700 FTIR with an ATR accessory. Preliminary experiments revealed that exposing conditioned PA-12 powder to two heat−cool cycles in the DSC did not alter the observed IR spectra. Therefore, samples were recovered from the DSC pans for subsequent ATR−FTIR analysis. Spectra were recorded at 4 cm^−1^ resolution and 125 scans, with 4-level zero filling and 0.482 cm^−1^ data spacing.

### 2.3. Fabrication and Conditioning of PA-12 Tensile Specimens

To characterise the mechanical properties of conditioned PA-12, plaques were hot pressed from PA-12 powder using a Moore E1127 Hydraulic Press. Then, 10 g of virgin PA-12 powder was placed in the centre of a polytetrafluoroethylene (PTFE) frame (18 cm × 18 cm × 0.022 cm) and sandwiched between two stainless steel plates. This was inserted between the pre-heated hot press platens, at 200 °C for 3 min, then pressed with a 10-tonne load for 3 min, at the same temperature. A slow cooling method under atmospheric conditions (~12 °C·minute^−^^1^), with no pressure applied, produced a plaque with thermal properties most similar to PA-12 powder.

Dog-bone tensile samples, with a gauge length of 28 mm, width 4 mm and thickness of ~0.23 mm were stamped from the PA-12 plaques using a cutting die. Thickness was measured using a micrometer screw gauge and taken from an average of 3 locations across the sample. Tensile samples were then stored under the same oven conditions as PA-12 powder (Section 2.2). Following removal from the oven, the specimens were tested on an Instron 5566 materials tester using a 1 kN load cell and 10 mm/s crosshead speed. Proprietary Instron Bluehill analysis software enabled measurements of yield strength (YS), ultimate tensile strength (UTS), fracture strength (FS), elongation at break (EAB) and Young’s modulus. For each parameter, an average was determined from six tensile samples per storage time to ensure reproducibility. Statistical analysis on the measured data was carried out using a simple *t*-test. We note that there are limitations to this aspect of the study. To create the plaques for mechanical testing, the powder material was exposed to one thermal cycle, but this is unavoidable.

### 2.4. Effect of Drying PA-12 Powder before Oven Conditioning

To determine the effect of water content on degradation, virgin PA-12 was dried in a desiccator for the following time periods (days): 4, 40, 100. Following this pre-treatment, “dried” PA-12 was annealed under identical oven storage conditions to virgin (“un-dried”) PA-12 (Section 2.2). Comparisons between the behaviour of virgin and dried PA-12 were made.

## 3. Results and Discussion

### 3.1. Characterisation of Virgin PA-12 Powder

#### 3.1.1. Sample Variability

**Figure 1:**
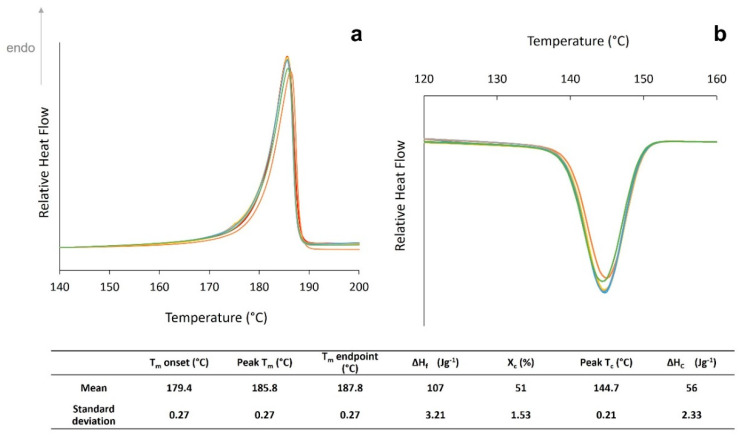
Virgin PA-12 powder sample variability for (**a**) melting behaviour and (**b**) crystallisation behaviour.

Un-conditioned, “virgin” PA-12 powder displays consistent and reproducible thermal properties, as shown by the insignificant sample-to-sample variability and low standard deviation values (Figure 1).

#### 3.1.2. Initial Assessment of Thermal Stability

**Figure 2:**
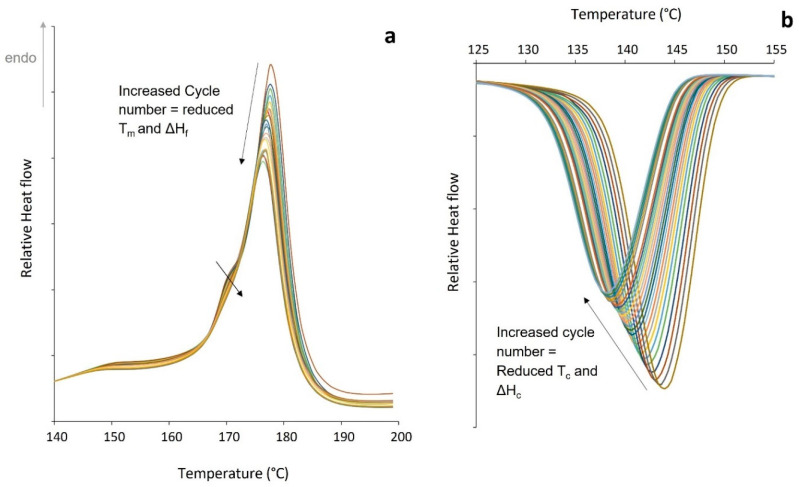
Change in (**a**) melting behaviour on heating and (**b**) crystallisation behaviour on cooling with repeated thermal cycling, to an upper temperature limit of 215 °C.

An initial assessment of the stability of the polymer was investigated by exposing virgin PA-12 powder to repeated thermal cycling. A sample was subjected to 25 heat–cool cycles, with a temperature range of 25 °C to 215 °C. With increased cycle number, both peak melting temperature (T_m_) and heat of fusion (ΔH_f_) reduced (Figure 2a). There was also a significant change in the crystallisation behaviour, as shown by reductions in peak crystallisation temperature (T_c_) and heat of crystallisation (ΔH_c_) (Figure 2b). This indicates a progressive hinderance to the crystallisation process and results in the observed changes in the subsequent re-heat. Repeated heating of PA-12 to high temperatures makes polymer chains highly mobile and more-reactive, which can result in linear chain growth via further polycondensation [1,2,12,13,14,20]. It can be envisaged that these longer polymer chains then exhibit hindered mobility due to increased chain entanglement, which restricts the formation of ordered crystalline regions [20]. Alternatively, some have suggested that cross-links can form within PA-12 [10,21], creating physical links between amorphous chains that could be described as tie-molecules [45], their effect being to hinder the crystallisation process. However, as the DSC is flushed with nitrogen, it is more likely that polycondensation causes the observed changes in melting and crystallisation behaviour.

Initially, a notable low temperature endotherm was apparent on the main melting peak (demonstrated by the small shoulder to the left of the main peak), but with increasing cycle number, the shoulder reduces in size and disappears (Figure 2a). There are two theories that could explain this behaviour. Some suggest that it represents the melting of thinner, less “perfect” crystals [14,46,47]. However, one can also interpret that the shoulder peak is caused by the reorganisation of polymer chains during heating, as a result of melt-recrystallisation, which stems from the metastability of the system [28,30,32,48]. With increased time spent above the melting temperature, further polycondensation and cross-linking hinders chain mobility and limits polymer reorganisation on heating, so the shoulder on the main peak diminishes.

**Figure 3:**
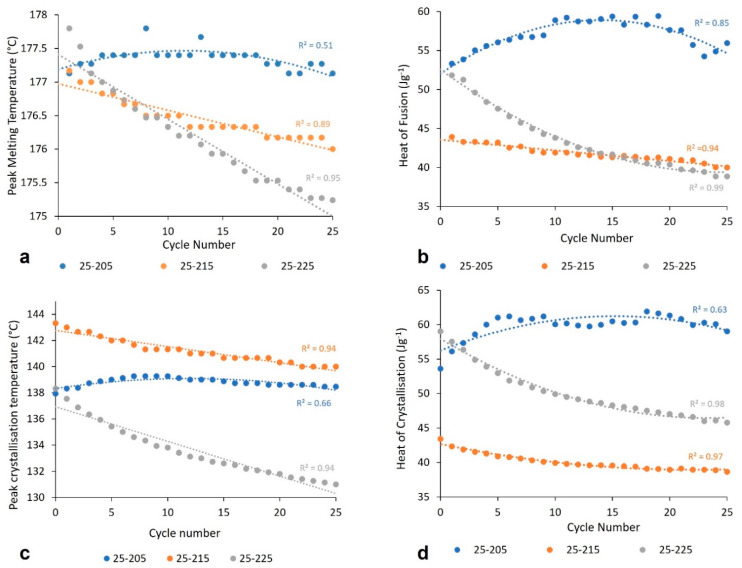
The change in (**a**) peak T_m_, (**b**) heat of fusion, (**c**) peak T_c_, and (**d**) heat of crystallisation with increased thermal cycling, with varied upper temperature limits.

This thermal cycling DSC experiment was repeated to investigate how the thermal stability of PA-12 may vary, when powder samples are repeatedly heated to different temperatures in the melt. On this occasion, two separate samples of virgin PA-12 were subjected to 25 heat–cool cycles, with a temperature range of 25 °C to 205 °C and 25 °C to 225 °C, respectively. Figure 3 indicates that the behaviour of the polymer depends on the upper temperature limit. When repeatedly heated to 205 °C, the melting and crystallisation behaviour of PA-12 shows what can conveniently be described as a parabolic response. This could be explained by a “self-seeding” process [49,50]. Although 205 °C is greater than the observed melting point, some thicker lamellae may not fully melt, which can result in residual lamellae. These small crystalline fragments persist into the melt and can act as nucleation sites which accelerate the re-crystallisation process on cooling [30,32]. Thus, as indicated by the blue trends in Figure 3, there is an initial increase in T_c_, ΔH_c_, T_m_, and ΔH_f_. However, with continued thermal cycling, the residual lamellae eventually fully melt, halting the “self-seeding” process, and a reduction in these parameters is then observed. As previously shown in Figure 2, with an upper limit of 215 °C, there is a progressive, albeit small, reduction in T_m_, ΔH_f_, T_c_, and ΔH_c_, which indicates some degradation in the form of further polymerisation and cross-linking. When heating to 225 °C, the polymeric sample is repeatedly exposed to a higher temperature, so the level of degradation is greater. Polycondensation and cross-linking are time and temperature dependant processes, and thus, they occur to a greater extent in the sample heated to 225 °C. As such, more significant reductions in T_m_, ΔH_f_, T_c_, and ΔH_c_ were observed, as indicated by the grey trend lines (Figure 3).

### 3.2. Oven Conditioning of Virgin PA-12 Powder

#### 3.2.1. Differential Scanning Calorimetry (DSC)

Over 336 h of oven storage, there appeared to be a marked time-dependency to the aging and degradation processes that were dominant over this timescale (Appendix A—Table A1). The melting and crystallisation behaviour of PA-12 powder showed two separate trends dependent upon storage time, and these are considered separately.

##### The First 100 h of Storage

**Figure 4:**
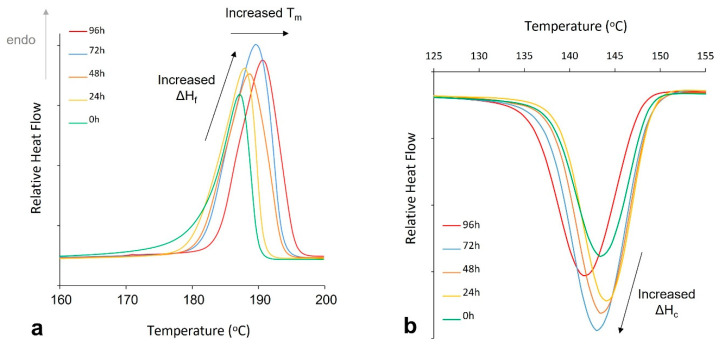
DSC first heat–cool showing the change in (**a**) melting behaviour and (**b**) crystallisation behaviour, for the first 100 h of oven storage.

After the first 100 h of storage, T_m_ increased by 4.2 °C. There was also a progressive increase in ΔH_f_ and ΔH_c_, while T_c_ displayed a slight reduction (Figure 4). Increases in T_m_ have previously been explained by polycondensation [10,13,17,19,20,35,39,40,41,42] and secondary crystallisation [10,20,21,40,46,48].

Polycondensation increases molecular weight; some suggest a higher temperature is therefore required to overcome the additional hydrogen bonding present in the lamellae of long polymer chains [10,20,21]. However, this process occurs exclusively in the amorphous phase and causes an increased number of entanglements, which hinders the crystallisation process [1,2,12,13,14,20]. As such, polycondensation alone would be expected to result in a larger reduction in T_c_, whilst ΔH_f_, a direct measure of crystallinity, would remain unchanged.

Secondary crystallisation and polycondensation could both occur simultaneously under the conditions of this study. Previous studies have shown that chain growth via polycondensation occurs primarily on thermally unstressed (virgin) PA-12 and, due to reduced availability of end-groups, is unable to continue at the same rate within aged powder [5,10,16,26,46]. As such, continued growth of T_m_ for 100 h provides strong evidence that secondary crystallisation, via lamellar thickening [10,37,40,47,48,51,52], also contributed to the observed changes. This is further supported by increased ΔH_f_ and a rise in ΔH_c_ which links to the previously explained “self-seeding” process and the formation of thicker lamellar structures [32].

##### Storage Times Greater Than 100 h

**Figure 5:**
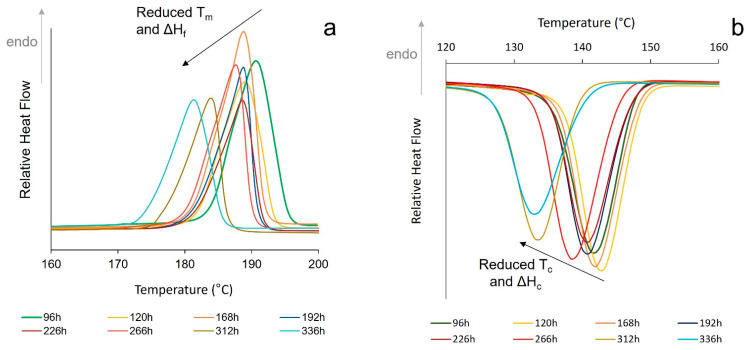
DSC first heat–cool showing the change in (**a**) melting behaviour and (**b**) crystallisation behaviour, for storage times greater than 100 h.

For storage times greater than 100 h, the initial trends reverse. After extended storage times, there is a significant decrease in T_m_ and ΔH_f_ (Figure 5a), as well as substantial reductions in T_c_ (Figure 5b). These changes provide evidence of degradation and, as storage time increases, degradative processes become predominant over previous aging phenomena. Prolonged oven storage provides suitable conditions for thermo-oxidative degradation as PA-12 powder is exposed to high temperatures, in the presence of oxygen, for extended time periods [5,10,16,24,37,52,53]. This results in rapid chain scission and significant reductions in molecular weight [21,36,48,52,53]. As such, melting point depression occurs due to the acidic degradation products of thermo-oxidation [53].

**Figure 6:**
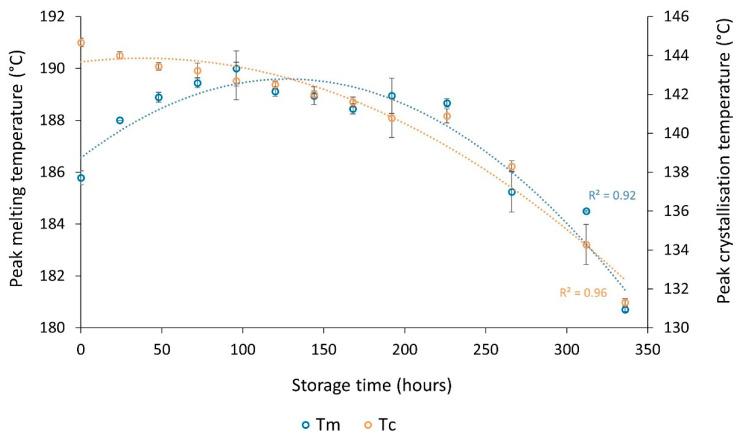
The overall change in T_m_ and T_c_ after storage of PA-12 at 170 °C for up to 336 h.

Thermo-oxidative degradation may also cause the decrease in ΔH_f_ and the accelerated reduction in T_c_, which both signify a delayed and diminished crystallisation process. Thermo-oxidation can result in oxidation products such as carboxylic acids, aldehydes, and imides [52]. These products have additional, bulky side chains which prevent polymer chains rearranging into an ordered structure and may explain the observed reduction in T_c_. As such less crystalline regions form during cooling, and lamellar structures are generally thinner, so T_m_ and ΔH_f_ also reduce. There is a strong relationship between the melting and crystallisation behaviour (Figure 6), emphasising that degradation is the underlying causation of the changes in thermal properties at extended storage times.

#### 3.2.2. Attenuated Total Reflection—Fourier Transform Infrared Spectroscopy (ATR-FTIR)

**Figure 7:**
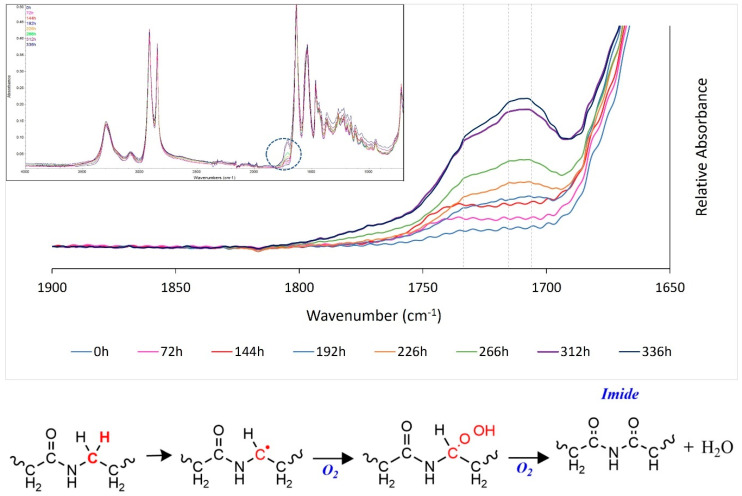
A full ATR-FTIR spectra for PA-12, with the carbonyl region magnified to show the development of a new band at 1700–1760 cm^−^^1^, with absorption maxima appearing at 1705 cm^−^^1^, 1715 cm^−^^1^, and 1733 cm^−^^1^, as indicated by the dashed grey lines. The chemical reaction resulting in the formation of imide bonds is displayed.

Infrared spectroscopy is a characterisation technique commonly used to investigate oxidative aging [48,52,54,55,56], but it has not been fully utilised in previous powder bed fusion (PBF) studies. In the current study, following extended oven storage (>150 h), a new absorbance band, containing various absorption maxima, began to form as a notable shoulder on the Amide I carbonyl peak (Figure 7). Similar behaviour has been seen previously within polyamides and is associated with thermo-oxidative degradation of PA-12 [48,52,54,55,56,57]. As such, the growth of this shoulder peak can be used as an indicator for the onset of degradation.

With increased storage time, three distinct absorption peaks became apparent (Figure 7). An increase in absorbance at 1733 cm^−^^1^ is associated with the formation of imide bonds, often used as the main marker of thermo-oxidative degradation [48,52,54,55,56,57,58]. Similar increases in absorbance occurred at 1715 cm^−^^1^ and 1705 cm^−^^1^, which are caused by other products of the polyamide oxidation cycle, such as carboxylic acids [48,52] and aldehydes [56,57], respectively.

The primary degradation pathway of polyamides involves oxidation of the methylene carbon adjacent to the amide nitrogen, resulting in the formation of an imide group from the amide (Figure 7) [24,54,55,57,58,59]. Storage of PA-12 powder at high temperatures, close to the melting point, accelerates this process [24,57]. The production of an imide group facilitates chain scission at the carbon–nitrogen bond [54,55,57], which creates a strongly destabilised polymer. Within polyamides this instability allows thermo-oxidative degradation to proceed without an induction period [58]. As such, once degradation becomes dominant it can accelerate with a low activation energy resulting in rapid reductions in molecular weight, as well as significant changes to the thermal (Section 3.2.1) and mechanical properties (Section 3.2.4) of the material.

#### 3.2.3. Relationship between Aging and Degradation Processes

**Figure 8:**
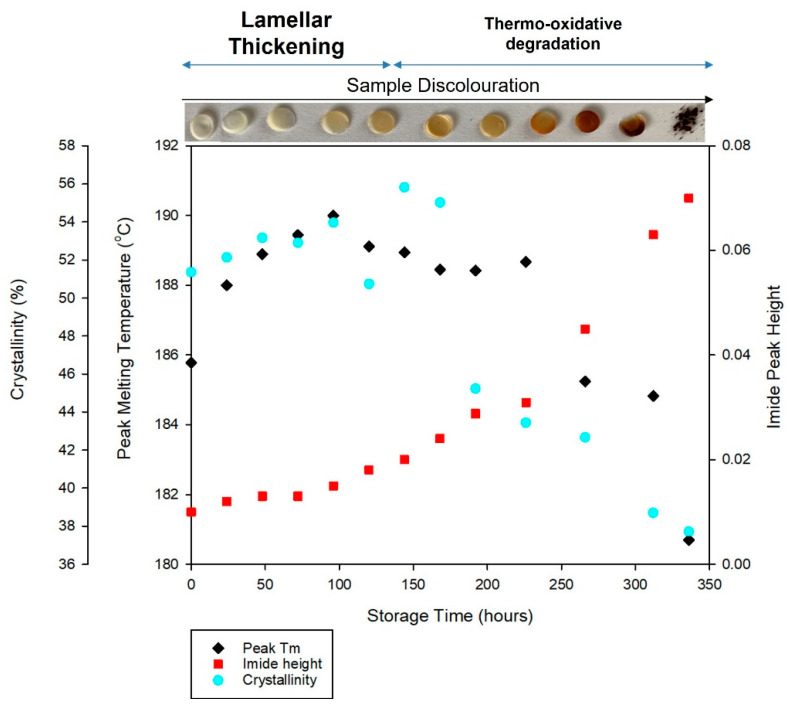
Correlation between changes in PA12 T_m_, crystallinity, imide peak height growth and sample discoloration, with storage time. Displays the relationship between polymer morphology and degradation.

Characterisation of PA-12 powder using DSC and FTIR provides evidence of multiple aging and degradation processes occurring during storage at 170 °C in air. Figure 8 indicates that there is an interplay between these aging phenomena and, across the full duration of storage time, the dominant aging process changes.

Initially, there is an increase in peak melting temperature (T_m_) and crystallinity (X_c_), which can be associated with lamellar thickening [10,20,21,40,46,48], yet there is an insignificant change to imide peak height. However, with extended storage beyond 150 h, there is a reduction in T_m_ and X_c_. This is closely correlated with a significant increase in imide peak height, indicating thermo-oxidative degradation [48,52,54,55,56,57]. The extent to which degradation occurs is exposed by the discolouration of the samples, whereby they changed from translucent to yellow, and then dark brown (Figure 8), as seen previously [51,52,54,56,57]. Lamellar thickening may still be occurring, but any effects on polymer morphology and thermal properties are masked by the ever-accelerating degradation process.

Furthermore, polymer morphology, particularly the percentage crystallinity of PA-12, influences the extent of degradation processes. Some have hypothesized that diffusion of oxygen and water cannot happen in crystalline regions of the material [37,55,58]. However, others suggest degradation occurs via a two-step process. Firstly, preferential attack of the more easily accessible amorphous chains takes place, before the degradation of crystalline regions, which coincides with polymer mass loss [60,61]. Regardless, chain scission due to thermo-oxidation (and hydrolysis, see Section 3.3) is thought to occur primarily in the amorphous phase. As such, with extended storage time, degradation of PA-12 is accelerated by the reduction in crystallinity, because there are less crystalline regions remaining to combat the intake of oxygen. This is exacerbated by an increased presence of acidic groups, which accelerate degradation through a process known as “autocatalysis” [62,63].

#### 3.2.4. Mechanical Properties of PA-12 Plaques

**Figure 9:**
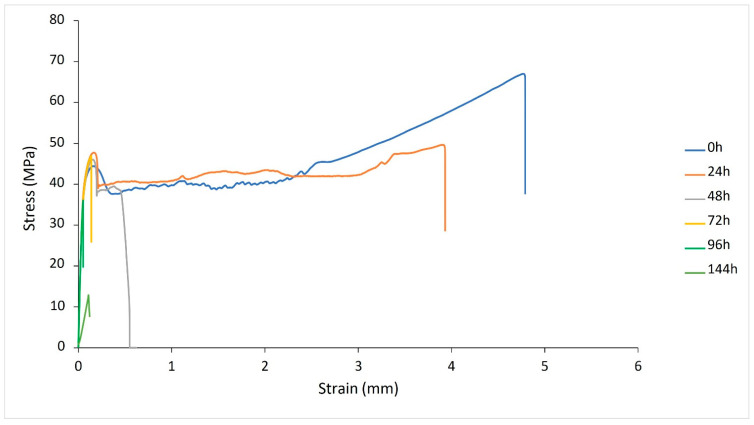
Stress–strain curves for PA-12 tensile samples as a function of storage time.

**Figure 10:**
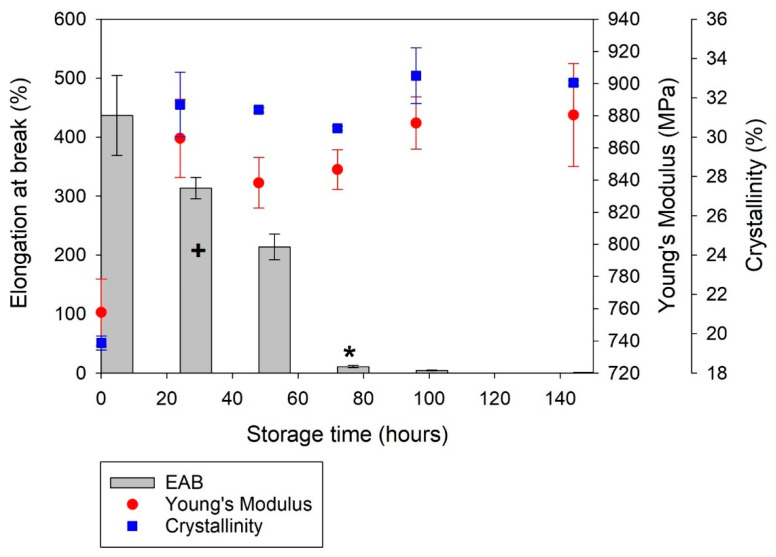
The change in elongation at break (EAB) Young’s modulus, and crystallinity of PA-12 plaques with increased storage time. + Significant change in EAB when compared to the 0 h sample (*p* < 0.05). * Very significant change in EAB when compared to the 0 h sample (*p* < 0.005).

There was a significant change in the mechanical properties of PA-12 following storage at 170 °C for an extended period. Un-conditioned PA-12 displayed ductile behaviour, but with increased storage time there was progressive embrittlement of the material until almost immediate fracture (Figure 9). All measures of strength exhibited similar behaviour; a gradual reduction observed for the first 72 h of storage, followed by a more rapid decrease (Table 2). There was an 81% reduction in ultimate tensile strength (UTS) between un-conditioned PA-12 and tensile samples stored for 144 h. Values of elongation at break (EAB) significantly reduce with increased storage time, with a corresponding rise in Young’s modulus (Figure 10). Un-conditioned PA-12 samples presented an EAB of 436.7% with a standard deviation of 67.9 (436.7 ± 67.9%). This value decreased to 213.7 ± 21.8% after 48 h of storage and 1.03 ± 0.3% after 144 h, demonstrating significant embrittlement of the material. This provides more evidence of severe degradation, so no further oven storage times were required.

As discussed in Section Storage Times Greater Than 100 h and Section 3.2.2, the conditions adopted in this study ultimately result in thermo-oxidative degradation and subsequently chain scission of PA-12 [10,21,24,37,52,53]. The observed changes in mechanical properties provide more evidence of oxidative degradation because reduced tensile strength is a strong indicator of decreasing molecular weight [64,65,66]. Within PA-12, degradation involves an auto-oxidation mechanism whereby hydrogen atoms are abstracted from the polymer backbone, causing a loss of amide groups, and a reduction in hydrogen bonding between adjacent polymer chains [22,24,54,58,59]. This contributes to a significant decrease in molecular weight [5,16,36,48], as well as substantial reductions in strength and EAB [24,37,54,55,57,59,67].

Degradation can cause a reduction in Young’s modulus [56,68], but in this case, within the first 24 h of oven conditioning, Young’s modulus increased by 109 MPa. Across the same period, a 12% increase in the crystallinity of PA-12 tensile samples was observed (Figure 10). For the duration of oven storage, the changes in Young’s modulus and crystallinity are linked, and both remain elevated relative to un-conditioned PA-12 plaques. This suggests that secondary crystallisation may have occurred in the conditioned tensile samples, albeit alongside degradation processes.

During oven storage, morphological and structural changes to PA-12 could be attributed to two different phenomena, lamellar thickening via thermal annealing [28,40,48] or chemi-crystallisation, which is initiated by degradation [36,37,38,48,52]. Due to the macromolecular nature of semi-crystalline polymer chains, they usually contain a high number of entanglements, which can limit further crystallisation [38]. However, during chemi-crystallisation, chain scission allows previously entangled amorphous sections to be released which permits further crystallisation via rearrangement of the smaller, and subsequently more mobile polymer chains [36,37,38]. Both secondary processes enhance the degree of crystallinity, reducing the volume fraction of the amorphous phase, which explains the observed reduction in EAB [36,37,69] and increase in Young’s modulus [48,69].

**Figure 11:**
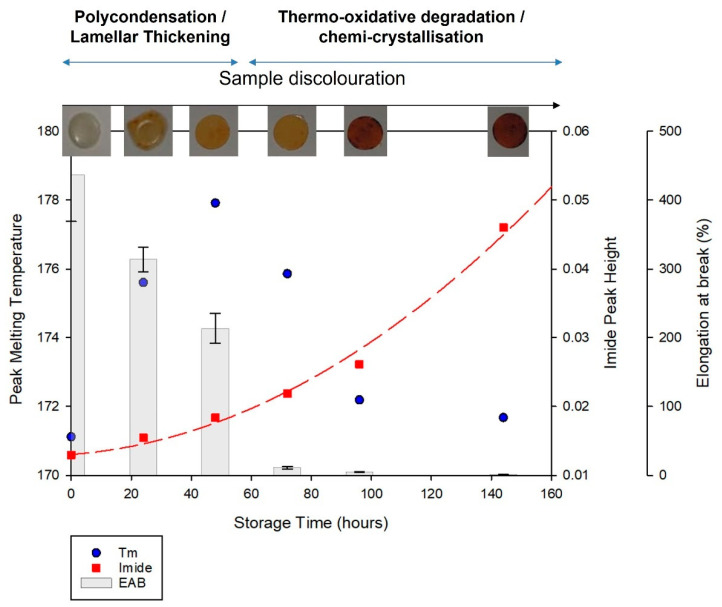
Relationship between PA-12 plaques thermal, chemical, mechanical, and optical properties as a function of storage time.

Characterisation of the PA-12 plaques using DSC and FTIR allows the aging behaviour of the plaques to be compared to that of the powder. Figure 11 displays the thermal, chemical, mechanical, and optical properties of PA-12 plaques and generally the observed behaviour is comparable to PA-12 powder (as shown in Figure 8). A similar initial increase in T_m_ occurs, and with extended storage time, PA-12 plaques show decreased T_m_ and increased imide peak height and sample discolouration. These changes indicate thermo-oxidative degradation, which also explains embrittlement of PA-12 [37,48,54,56]. Although the observed trends are similar, the key difference is that degradation occurs at an accelerated rate within PA-12 plaques, which are less stable, and melt at a lower temperature than the powder samples. This is due to the unavoidable degradation that occurs during hot-pressing. As such, after only ~150 h of storage, the samples were so heavily degraded that oven conditioning was discontinued. Similarly, reprocessing altered the changes in PA-12 crystallinity. Accelerated degradation, as well as storage conditions being closer to the T_m_ of the reprocessed material, allows chemi-crystallisation to occur within PA-12 plaques, resulting in increased crystallinity.

### 3.3. Oven Conditioning of Pre-Dried PA-12 Powder

As shown in Section 3.2 degradation processes alter the thermal properties of PA-12, resulting in significant changes to the melting and crystallisation behaviours. However, the presence of amide groups and hydrogen bonding renders polyamides polar and highly hydrophilic, so PA-12 is also sensitive to chain scissions in the presence of water and moisture, through hydrolysis [36,48]. Water molecules mobilise polymer chains which increases the rate of diffusion of oxygen into the polymer [55,59,70].

**Figure 12:**
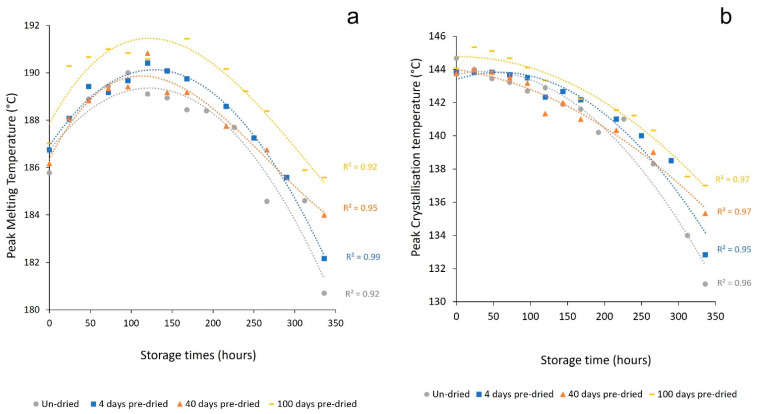
The change in (**a**) peak melting temperature and (**b**) peak crystallisation temperature with storage time, as a function of time spent drying in a desiccator prior to oven conditioning. Error bars were removed for clarity of the data trends, whilst all standard deviation values for these datasets was <1.0 °C, so considered insignificant in relation to the observed trend.

**Figure 13:**
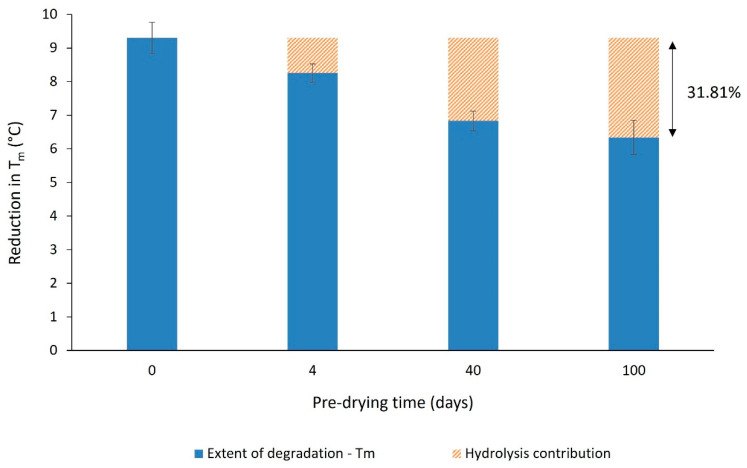
The reduction in melting temperature of PA-12, as a function of pre-drying time. Changes in T_m_ were calculated from the highest value during oven storage to the final value observed after 336 h.

To limit the possibility of hydrolysis, unconditioned PA-12 powder was dried in a desiccator, without elevating the temperature. Dried samples were then conditioned in the oven (as explained in Section 2.2) and compared to un-dried PA-12. With increased pre-drying time, melting point depression diminished (Figure 12a), whilst the change in T_c_ was also lowered (Figure 12b). Similarly, the maximum T_m_ observed during oven conditioning is deferred to later storage times, which suggests the onset of degradation is delayed. After 100 days pre-drying, the reduction in T_m_ is only 6.3 °C, compared to a 9.3 °C decrease in un-dried PA-12 (Figure 13). This provides evidence that thermo-oxidative degradation is accelerated by moisture within PA-12, leading to more rapid chain scissions and subsequently more significant reductions in molecular weight [36,48,56,59]. As a result, pre-drying PA-12 before exposure to heat and oxygen assists water removal, which reduces the extent of polymer degradation.

**Figure 14:**
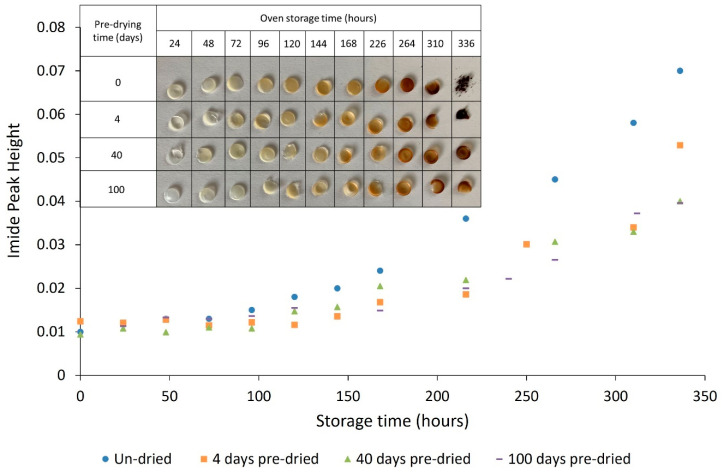
The effect of drying PA-12 powders before oven conditioning on the growth of the imide peak and sample discolouration.

The effect of pre-drying PA-12 before oven conditioning is further supported by FTIR data. Figure 14 displays the change in imide peak height as a function of storage and pre-drying time; the growth of the imide band is significantly greater in un-dried PA-12. With increased pre-drying time, there was a consistent reduction in imide growth, emphasising decreased thermo-oxidative degradation. After 336 h of oven storage, un-dried PA-12 has an imide peak height of 0.07, and with 100 days of pre-drying, imide peak intensity is only 0.039. This highlights the effect of hydrolysis on the extent of PA-12 degradation.

Another indicator of thermo-oxidative degradation is sample discolouration [51,52,54,56,57], which appears linked to imide growth (Figure 14). With increased storage time, all samples change from translucent to yellow to dark brown. However, as shown by the table in Figure 14, sample discolouration becomes less pronounced as pre-drying time is increased, with the most notable differences appearing in the samples stored for greater than 200 h. This provides further evidence that pre-drying PA-12 can reduce degradation.

## 4. Conclusions

This investigation included a unique analysis of conditioned PA-12 samples using FTIR, a technique rarely utilized in PBF literature, in addition to DSC. Characterisation indicated that across 336 h of oven conditioning, there were two separate, time-dependant trends, which suggested an interplay between the multiple aging processes occurring during PBF.

Initially, DSC showed a 4.2 °C increase in T_m_, as well as progressive increases in ΔH_f_ and ΔH_c_, but only a 1.8 °C reduction in T_c_. Across the same period of storage, FTIR spectra displayed an insignificant change in imide peak height. As such, due to the increase in crystallinity over the first 100 h, secondary crystallisation appears to be the dominant aging process over this period. However, between 100 h and 336 h of storage, there was a 9.3 °C reduction in T_m_ and a 11.8 °C decrease in T_c_. These changes are closely correlated with an accelerated increase in imide peak absorbance and significant sample discolouration. Hence, with extended storage times, thermo-oxidative degradation became dominant over other aging processes. The mechanical behaviour of PA-12 plaques supports the trends observed in PA-12 powder. Un-conditioned PA-12 displayed ductile behaviour, but with increased storage time, there was progressive embrittlement of the material and significant reductions in strength. The loss of mechanical properties is likely a consequence of chain scission and subsequent reductions in molecular weight as a result of thermo-oxidation. Samples of virgin PA-12 powder were pre-dried in a desiccator to examine the effect of hydrolysis on PA-12 degradation. With 100 days pre-drying, the reduction in T_m_ after 336 h of storage was 3 °C less than un-dried PA-12. Similarly, pre-drying saw a decrease in the presence of imide bonds and reduced sample discolouration. These differences show that thermo-oxidative degradation is accelerated by moisture present within PA-12.

These results illustrate an interaction between the multiple aging and degradation processes which can occur when PA-12 is exposed to conditions found within MJF. Through a combination of characterisation techniques, the dominant aging process across different periods of storage was quantified. As well as adding to the research community, this improved understanding could be utilised by the AM industry. The current use of set, arbitrary refresh ratios could be addressed to help develop a more sustainable and cost-effective recycling strategy in the future.

## Figures and Tables

**Table 1:** Experimental conditions adopted for thermal analysis.

Experiment	Sample Mass (mg)	Heating/Cooling Rate (Kmin^−1^)	Heating Range (°C)	No. of Heat−Cool Runs
Sample variability	6 ± 0.5	10	25–220	2
Thermal stability		40	25–205, 25–215, 25–225	25
Post-oven Conditioning		10	25–220	2

**Table 2:** The variation of mechanical properties with storage time.

Storage Time (Hours)	Yield Strength (MPa)	Ultimate Tensile Strength (MPa)	Fracture Strength (MPa)
0	28.1 ± 1.2	62.1 ± 8.2	59.8 ± 7.2
24	27.1 ± 0.9	49.2 ± 2.8	44.8 ± 4.0
48	25.4 ± 0.6	47.1 ± 3.9	46.1 ± 4.7
72	25.3 ± 1.4	44.8 ± 2.0	44.0 ± 2.3
96	25.2 ± 1.8	34.6 ± 1.1	34.6 ± 1.1
144	11.6 ± 2.4	11.6 ± 2.4	11.6 ± 2.4

## Data Availability

Not applicable.

## Appendix A. DSC Data

**Table A1:** Experimental data at all storage times for PA-12 powder, whereby the values shown are taken as an average from 3 repeats.

Storage Time (Hours)	Peak T_m_ (°C)	T_m_ Endpoint (°C)	ΔH_f_ (Jg^−1^)	Crystallinity (%)	Peak T_c_ (°C)	ΔH_C_ (Jg^−1^)
0	185.8 (±0.27)	187.8 (±0.27)	107 (±3.2)	51 (±1.5)	144.7 (±0.21)	54 (±2.3)
24	188.0 (±0.00)	190.9 (±0.58)	109 (±4.2)	52 (±2.1)	144.0 (±0.13)	56 (±3.5)
48	188.9 (±0.11)	193.6 (±0.14)	111 (±2.9)	53 (±1.4)	143.4 (±0.11)	59 (±2.3)
72	189.4 (±0.11)	193.4 (±0.43)	111 (±3.1)	53 (±1.5)	143.2 (±0.22)	60 (±2.7)
96	190.0 (±0.28)	194.5 (±0.42)	113 (±2.8)	54 (±1.4)	142.7 (±0.39)	58 (±1.7)
120	189.1 (±0.11)	192.8 (±0.38)	106 (±0.8)	51 (±0.4)	142.9 (±0.11)	54 (±1.8)
144	188.9 (±0.20)	192.8 (±0.28)	117 (±7.0)	56 (±3.4)	141.9 (±0.11)	62 (±2.8)
168	188.4 (±0.08)	192.4 (±0.21)	115 (±0.9)	55 (±0.4)	141.6 (±0.10)	59 (±1.2)
192	188.4 (±0.39)	190.8 (±0.70)	95 (±7.3)	45 (±3.5)	140.2 (±0.67)	48 (±4.1)
226	188.7 (±0.09)	191.3 (±0.38)	91 (±6.2)	43 (±2.9)	141.0 (±0.17)	48 (±3.5)
266	184.8 (±0.45)	187.1 (±0.63)	107 (±4.7)	51 (±2.2)	138.3 (±0.17)	58 (±0.1)
312	184.6 (±0.02)	187.5 (±0.00)	81 (±0.7)	39 (±2.5)	134.0 (±0.49)	47 (±0.2)
336	180.7 (±0.04)	184.5 (±0.79)	79 (±7.2)	38 (±3.5)	131.1 (±1.24)	49 (±0.9)
